# Quantifying the alignment error and the effect of incomplete somatosensory feedback on motor performance in a virtual brain–computer-interface setup

**DOI:** 10.1038/s41598-021-84288-5

**Published:** 2021-02-25

**Authors:** Robin Lienkämper, Susanne Dyck, Muhammad Saif-ur-Rehman, Marita Metzler, Omair Ali, Christian Klaes

**Affiliations:** Department of Neurosurgery, University Hospital Knappschaftskrankenhaus Bochum GmbH, Ruhr-University Bochum, Bochum, Germany

**Keywords:** Brain-machine interface, Spinal cord injury

## Abstract

Invasive brain–computer-interfaces (BCIs) aim to improve severely paralyzed patient’s (e.g. tetraplegics) quality of life by using decoded movement intentions to let them interact with robotic limbs. We argue that the performance in controlling an end-effector using a BCI depends on three major factors: decoding error, missing somatosensory feedback and alignment error caused by translation and/or rotation of the end-effector relative to the real or perceived body. Using a virtual reality (VR) model of an ideal BCI decoder with healthy participants, we found that a significant performance loss might be attributed solely to the alignment error. We used a shape-drawing task to investigate and quantify the effects of robot arm misalignment on motor performance independent from the other error sources. We found that a 90° rotation of the robot arm relative to the participant leads to the worst performance, while we did not find a significant difference between a 45° rotation and no rotation. Additionally, we compared a group of subjects with indirect haptic feedback with a group without indirect haptic feedback to investigate the feedback-error. In the group without feedback, we found a significant difference in performance only when no rotation was applied to the robot arm, supporting that a form of haptic feedback is another important factor to be considered in BCI control.

## Introduction

The main goal of Brain Computer Interfaces (BCIs) is to improve severely paralyzed (e.g. tetraplegic) patients’ quality of live by decoding their movement intentions from neural data and giving them control over robotic devices. Invasive recording techniques allow to record motor imagery signals with high enough precision to decode many degrees of freedom directly from the motor system^1–3^. However, motor performance of patients using a state-of-the-art invasive BCI are still far away from healthy people’s performances, which limits the potential clinical use of BCIs. We consider this difference in motor performance the system error of a BCI setup. This is the target variable for BCI development, as a (hypothetical) perfect BCI would allow its user to perform on par with healthy subjects. The system error of a BCI system therefore limits the best possible motor performance a patient could achieve using it, which makes lowering the system error the key objective to improve the clinical use of BCIs. We propose that three major error-sources contribute to the system error: the decoding error, the alignment error and the feedback error. While the decoding error is arguably the most impactful source of error, the main focus of this work is to investigate the alignment and feedback error independent from each other and from the decoding error.

The decoding error describes the difference between a patient’s movement intention and the movement intention decoded from the recorded brain signals. Such a difference emerges when the recorded information is insufficient or because of decoder errors.

Alignment error emerges when the BCI’s end effector is misaligned relative to the participant’s natural arm. A robotic arm in BCI experiments is usually positioned so that it cannot reach and accidentally injure the patient^4,5^. Controlling a robot arm that is moved aside and is sometimes rotated relative to the patient requires patients to consider this distance in their movement planning and adapt to it. Since similar studies investigating motor performance in a 2D tracing task^6^ and control of rotated tools^7,8^, have shown a performance decrease caused by visuomotor transformations, we propose that the end-effector misalignment in BCIs also introduces a source of error. Whether such an error exists and how big it is compared to other error sources mentioned remains widely unknown and is the core question we address in this work.

A BCI system’s feedback error is the contribution of missing somatosensory feedback to the overall performance. In recent BCI-experiments, functional stimulation of the primary somatosensory cortex was used to provide artificial somatosensory feedback to both non-human primates^9^ and to tetraplegic patients^10,11^ to improve performance by reducing the feedback error. Similarly, feedback strategies targeting the peripheral nervous system have been shown to be another promising way to restore sensory feedback^12,13^. For this work, we investigated the influence of indirect haptic feedback—as a proxy for somatosensory feedback that paralyzed patients are missing—on performance in a shape-drawing task. The indirect haptic feedback is caused by the contact of the controller with the table surface when performing the drawing task in VR. We compare two groups of participants: one group receives indirect haptic feedback while the other does not, i.e. the physical table is removed in this case but the table in VR is still visible. Our indirect haptic feedback group therefore simulates a scenario where participants receive somatosensory feedback while actively controlling the end-effector.

The decoding error can be studied for the most part independently from the other error sources, for example by comparing different decoding algorithms using pre-recorded datasets^14–16^ or by using more abstract end effectors (i.e. computer cursors) that rely less on feedback and alignment than robotic limbs^17^. However, the feedback- and alignment error cannot easily be studied in isolation, as they require patients to control an end-effector, which in turn requires decoding. Results obtained using both a decoder and a robotic end-effector therefore represent a combination of decoding, alignment and feedback error and thus do not allow a precise evaluation of a single error source.

In this paper, we describe a method to study feedback- and alignment error in isolation. Healthy study participants with no sensorimotor dysfunctions that use perfectly tracked controllers in VR can be thought of as patients with motor dysfunctions using a perfect decoder in VR. The highly precise positional tracking of devices in VR allows us to project the movement of study participants onto a misaligned end-effector (in our case a robotic arm) simulating a situation that is similar to typical BCI-experiments in which a patient controls such a robotic arm. Our model therefore provides a one-to-one translation of movement intentions into end-effector movements, comparable to that of a patient using a perfect decoder. In this scenario, we then investigate whether the misalignment of the end-effector and the presence of indirect haptic feedback would still lead to a loss of motor performance compared to healthy subjects, and, if it does, how big this loss of performance is. Additionally, we compare two different control schemes to investigate whether the performance differs when the movement endpoint is translated into a coordinate frame rotated with the end-effector as compared to a Cartesian-endpoint control scheme, where the end-effector kinematics are not rotated. We use a shape-drawing task in virtual reality, a commonly used task in BCI experiments, that allows us to assess and compare motor performance under different conditions. Our data shows the effects of different robot-arm positions relative to the participant on motor performance, but also the possible gain from eliminating the alignment error altogether, for example by using an exoskeleton or functional electrical stimulation^18,19^, as these techniques would allow patients using a BCI to control their natural arm.

## Methods

The task we used in this experiment is similar to the mirror drawing task, a widely used neuroscientific method to assess motor performance and learning. In this study, however, we perform the task in a virtual environment using VR, and rather than observing their movements mirrored, the virtual environment allows us to freely adjust the visual feedback study participants receive (described below).

### Virtual reality hard- and software

All experiments were performed using an HTC Vive Pro (HTC Corporation, Taoyuan, Taiwan). The setup contained a virtual reality headset, controller and an additional tracker. The virtual environment was created using Unity3D, version 2018.2.1f1 (Unity Technologies, San Francisco, USA). Headset, controller, and the additional tracker were tracked in six degrees of freedom with a frequency equivalent to the virtual environment’s framerate. That framerate is subject to load-dependent variance. The mean framerate of all trials was 79 ± 5 (mean ± SD) frames per second. The highest framerate measured in a single trial was 89 frames per second, the lowest 64 frames per second. According to the manufacturer, the tracking system works at sub-millimeter precision. Study participants received a short introduction about virtual reality upon arrival and were given time to accustom themselves with the devices.

The VR-environment allowed us to study participant’s performance independent of any decoding errors. Using the hand tracking system provided by the VR hardware, we were able to simulate a BCI-setup with an “ideal decoder”, where participant’s movement intentions were translated into VR at sub-millimeter precision and no noticeable lag.

### Experimental design

Participants started the experiment seated in front of a table. In VR, a virtual table of similar size was presented with a canvas of approximately 88 × 44 cm size on it. The edges of the canvas were marked by a black outline. The virtual environment was built to resemble the lab room the experiments were performed in.

Participants held the controller, which appeared as a pen of similar size in VR, in their right hand. The tip of the pen was aligned with the bottom of the controller. By touching the canvas with the tip of the virtual pen, participants were able to “draw” on the canvas. We also used an additional tracker (Vive tracker, HTC Corporation, Taoyuan, Taiwan), a device that uses the same tracking system as the controllers and the headset and therefore allows to track the position and rotation of an additional object. It was placed on the table and used to align the virtual table’s height with that of the real table. This way touching the real table with the bottom of the controller was aligned with the tip of the virtual pen touching the canvas in VR and created haptic feedback similar to a real pen touching a table.

**Figure 1:**
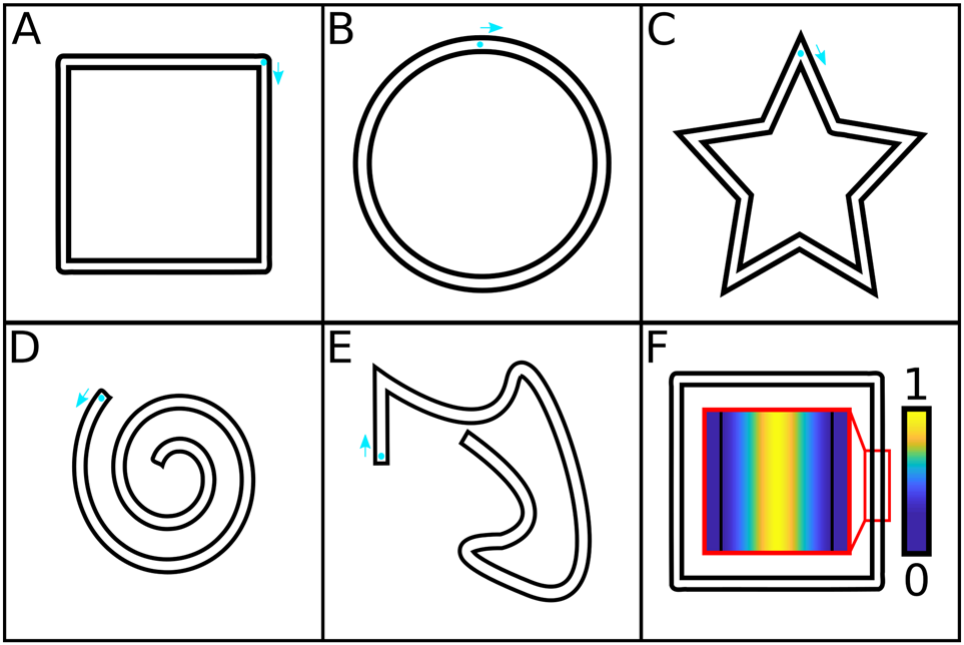
The shapes used for the shape drawing task (**a**–**e**). For every shape, the starting position is marked with a blue dot. Additionally, a blue arrow points from the starting position in the starting direction. Sub figure f shows an example of the scoring mechanism. An exemplary piece of the square shape (shown in **a**) is magnified in the red box and the scoring weights are shown color-coded as described in the text. Yellow indicates a higher score, blue a lower score.

Each trial, the virtual canvas showed the outline of a shape (the “target shape”) as black lines with a space of 1.25 cm in between (compare Fig. 1). Participants were instructed to draw on an imagined center-line between the two outline borders. Participants had to start drawing from a starting position marked with a blue circle. The starting position was fixed for each shape. Additionally, an arrow indicated the starting direction. The target shapes used in the experiment were a square (Fig. 1a), a circle (Fig. 1b), a five-point star (Fig. 1c), a spiral (Fig. 1d) and a custom-made asymmetrical shape (Fig. 1e). We chose these shapes to make sure that different difficulties are represented in the test. Additionally, we ensured that straight segments (e.g. in the square shape), segments with constant curvature (like in the spiral and circle) and sudden changes in drawing direction (corners as in the star or square shape) are represented.

Following each trial, study participants received feedback about their completion time. A timescale-gauge was shown above the virtual canvas. The gauge had a “green zone” (11.5–14.5 s completion time), a “yellow zone” (10–11.5 and 14.5–16 s) and a “red-zone” (more than 16 s or less than 10 s). A white line was shown indicating the last trial’s completion time on this scale. Completion times lower than 7 s or higher than 19 s where shown at the lower or upper end of the gauge. The time feedback was meant to ensure that study participants moved at a similar speed, even under conditions of varying difficulty. If a study participant repeatedly failed to stay in the green or yellow zone, they were reminded to try to stay in the “green zone” in between trials. The time limit was included to ensure that participants would not take more time for the more difficult conditions, potentially keeping the precision constant, but rather keep the completion time approximately constant.

To familiarize the study participants with virtual reality, the drawing mechanism and the time limit, the first five trials were done with the outline of an upper-case “M”. This target shape was not used later in the experiment and these five trials were not included in the analysis.

### Robot arm orientations

**Figure 2:**
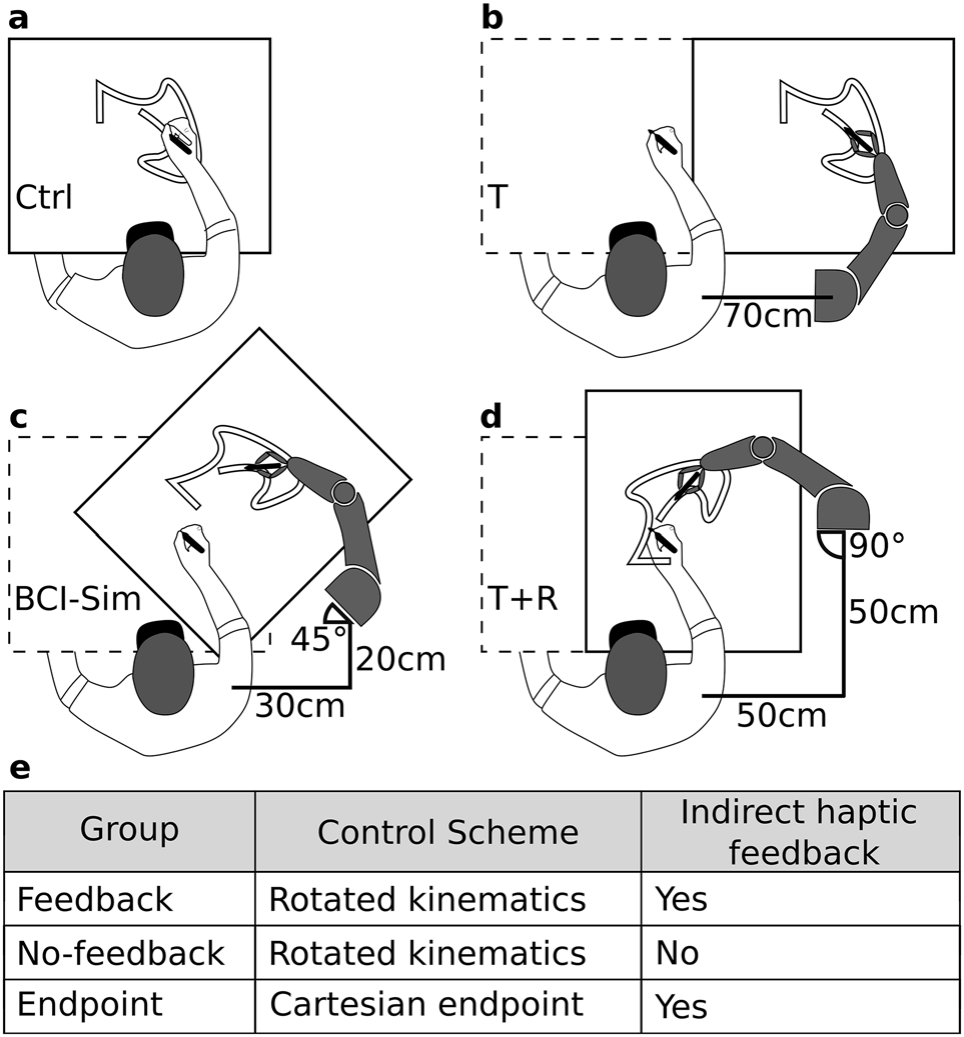
Schematic overview for the different conditions. Ctrl is shown in a, translation in b, BCI-Sim in c and the T + R condition in d. For the latter three conditions, the positioning of the robot arm relative to the participant’s shoulder are presented in the figure (the distances shown are not to scale). The virtual table’s position is marked with a black outline. The different combinations of haptic feedback and control schemes are shown in **e**. The with-feedback group performed the experiment with a real table matching the virtual table’s height. The real table’s position remained unchanged and is marked with dashed lines (for all conditions except control).

After the five training trials, participants performed four blocks for each of the four conditions, resulting in a total of 16 blocks per participant. The four conditions were: Control (Ctrl), translation (T), BCI-simulation (BCI-sim) and translation plus rotation (T + R). A schematic overview about the conditions is shown in Fig. 2.

In all conditions, participants had an avatar-body in virtual reality. The avatar body was in a seated position and its lower body remained motionless throughout the experiment. In the control condition (Fig. 2a), the virtual avatar’s right hand followed the controller movements and the corresponding arm joints were determined using inverse kinematics (Humanoid Control, Version 2.0.6, Passer VR, Hummelo, Netherlands). The virtual pen’s position matched the real-world position of the controller. The realistic visual feedback and the alignment of the avatar’s arm with the participant’s own arm therefore resembled a healthy person’s situation.

In all other conditions, the avatar’s arms remained motionless and were placed in the avatars lap. Instead, a robot arm resembling the MPL (Modular Prosthetic Limb) appeared in the room holding the pen. The original MPL was designed by the Johns Hopkins University Applied Physics Laboratory, which also provided a 3D model of the MPL. Depending on the condition, the arm was translated and/or rotated relative to the participant’s position and carried out the participant’s movements according to the control scheme used (see section “Control schemes” below). The table and the canvas were translated and rotated in the same way as the robot arm, ensuring that the drawing surface always remained directly in front of the robot arm (compare Fig. 2).

For the translation condition (Fig. 2b), the robot arm was placed 70 cm to the right of the participant’s right shoulder with no rotation applied, therefore facing the same direction as the participant. In the BCI-simulation condition (Fig. 2c) the robot arm was placed 30 cm to the right and 20 cm in front of the participant’s shoulder. Additionally, the arm was rotated by 45 degrees counterclockwise. In the translation plus rotation condition (Fig. 2d), the robot arm was placed 50 cm right and 50 cm in front of the participant’s shoulder and rotated by 90 degrees counterclockwise.

**Figure 3:**
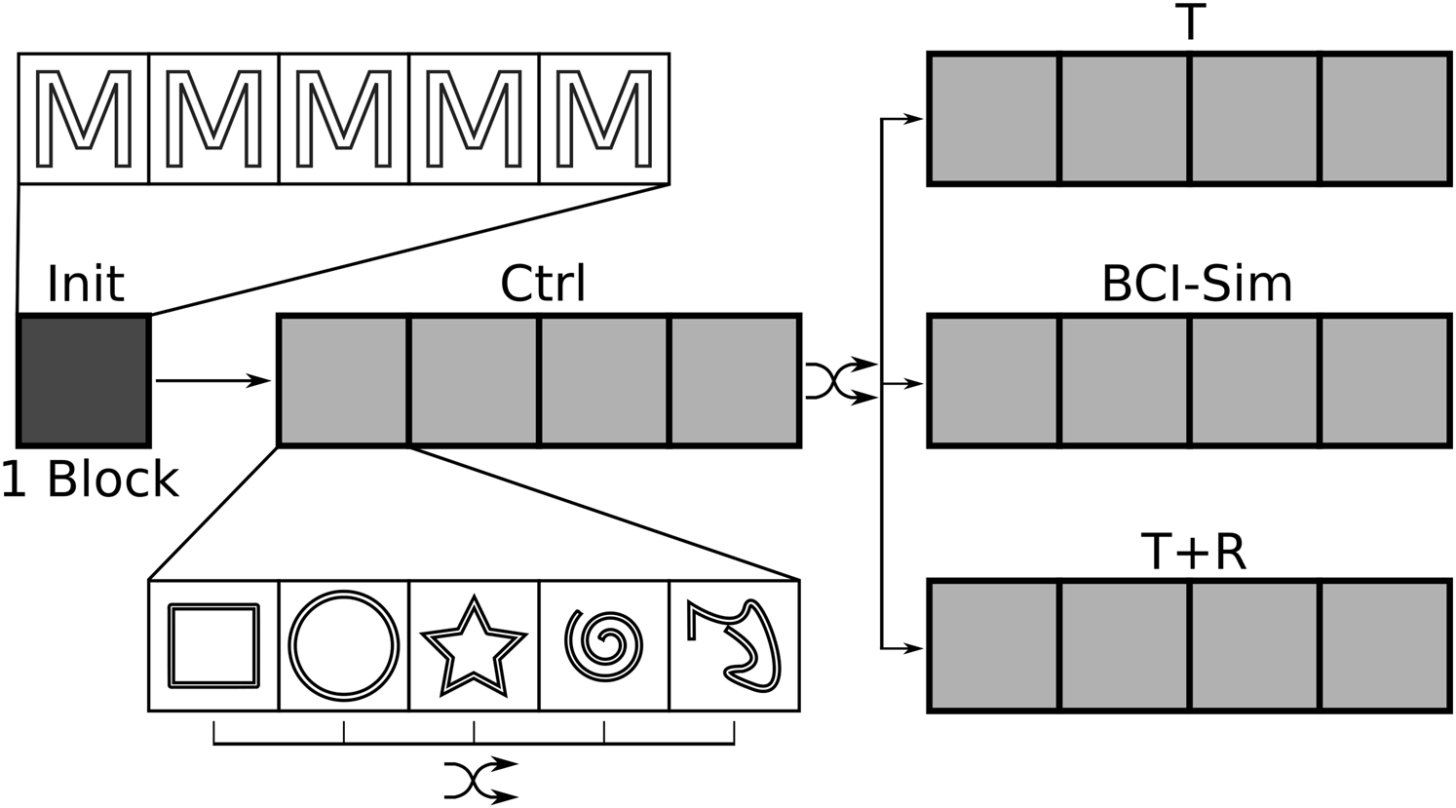
A schematic overview about the experimental procedure. Participants did five initial trials (one block) with an "M" target shape that was not used in the remaining experiment. After that, all participants did four blocks under control condition (CTRL). Each block contained one trial for each of the five target shapes in randomized order. The order of the target shapes was re-randomized every blocks. After the control condition, four blocks under one of the experimental conditions (T, BCI-Sim or T + R, chosen randomly) were done. The next four blocks were done under one of the two remaining conditions (again chosen randomly), followed by four blocks of the last condition. The order of the target shapes was re-randomized every block.

Participants performed four consecutive blocks in each condition. In every block, each of the five target shapes occurred once in a random order, resulting in 20 trials per condition. After the initial testing trials, all participants performed the control condition first. The remaining three conditions were performed in random order. An Overview about the experimental procedure is shown in Fig. 3.

### Feedback conditions

While the initial training trials were identical for all study participants, three groups of participants performed different versions of the task (compare Fig. 2e): the first group did the remaining experiment with the real table still being present, while for participants of group two the real table was removed after the initial five trials described above. However, movements of the second group were still constrained by the virtual tabletop surface, so that in both cases the virtual environment, including the virtual tables position and height, remained unchanged. This was done to investigate the performance of study participants that no longer received indirect haptic feedback when they touched the table with the hand-held controller and to compare their performance to participants that did. In the following, the first two groups will be referred to as the “feedback” and “no feedback” group, respectively. The third group (called the “endpoint” group) performed the experiment with the real table present, but using a different control scheme, as described in the following section (compare Fig. 2e).

### Control schemes

For participants in the feedback and no-feedback group, the virtual pen appeared at a position that had the same relative distance to the virtual robot arm as the actual controller had from the participant’s shoulder position. Their movements were therefore carried out in a relative coordinate frame centered on the robot arm. In contrast, the movements of participants in the endpoint group resembled a global cartesian endpoint control scheme: The coordinate system they moved in was centered around the center of the drawing surface and never rotated relative to the participant.

Due to the lack of any rotation in the translation condition, this condition would have been identical for the feedback group and the endpoint group. For the endpoint group, the translation condition was therefore changed to the same position as in the BCI condition (30 cm right, 20 cm forward), but without the rotation. This allows for a direct investigation of whether robot limb rotation still affects motor performance when participants used the endpoint control scheme, where the control signals are not rotated.

### Scoring

To compare the study participants’ performances, we used a scoring algorithm that assigns a number between zero and one to each tracking point of the data. The score of all tracking points was computed as a function of their distance from the optimal trajectory in the center of the target shape outline. The function used was a gaussian centered on the optimal trajectory with a standard deviation of 10. The gaussian was cut off at a range of 1.76 cm near the optimal trajectory. All point beyond this range were assigned a score of zero. An example of the scoring mechanism is shown in Fig. 1f.

### Scoring per segment

To compare a participant’s performance on certain segments of a target shape, we computed 100 equally spaced points along the optimal path (the path directly in the center of the target shape outline). Each of these points represents a segment of the target shape. A segments’ score was defined as the mean score of all tracking points in a trial that lie in a radius of 1.3 cm around the optimal point representing that segment. If no tracking point of a trial lied within this radius, it was assigned a score of 0. Segments’ scores were calculated on a per-trial basis. Since the circle radius of 1.3 cm is bigger than the distance between the optimal points, the circles representing segments overlap. Therefore, consecutive segment mean scores are not statistically independent.

### Study participants

A total of 38 subjects participated in the study reported in this paper. Three of them had to be excluded from analysis for technical reasons, leaving 35 participants (15 females, 20 males) for further analysis. The participants were split into three groups: the feedback group contained 13 participants (8 male), the no-feedback group contained 12 participants (8 male) and the endpoint group contained 10 participants (4 male).

All participants were between 18 and 47 years old and had normal or corrected-to-normal vision. Only right-handed participants were accepted for the study. Participant’s handedness was confirmed using the Edinburgh Handedness Inventory^20^. Participants received monetary compensation for participating in the study and gave written informed consent. All experiments were approved by the local ethics committee of the Ruhr-Universität Bochum and were performed in accordance with the declaration of Helsinki.

### Questionnaires

After the experiment, participants were asked to fill out a questionnaire and rate the difficulty of different aspects of the experiment. Firstly, they were asked to rate the five target shapes in difficulty, assigning the numbers one to five to the shapes in ascending order of difficulty. No duplicate answers were allowed. In the second and third question participants had to select which condition (except control) they considered the hardest and the second hardest. These questions give us insights in the participants’ subjective experience, while the scoring mechanism described above gives us an objective measure of a participant’s performance. These measures can then be used for comparisons or to find whether the optimal end-effector positioning varies individually. Since some people experience motion sickness in VR scenarios, participants were asked whether they felt dizzy at any time during the experiment. This information allows us to assess the influence of the virtual reality setting on participants’ performances. If a participant had significant motion sickness while performing the task their data would be excluded from analysis. However, no participant reported significant motion sickness and therefore no participants have been removed from further analysis because of this. Finally, participants rated the tracking quality (“How well did the controller follow your hand- and arm movements?”) on a five-point Likert scale. That way, we can evaluate whether participants had problems with the VR hardware that affected their performance.

### Statistical analyses

Three types of statistical analyses were used in this work. Firstly, to determine whether differences between the conditions of one group (feedback, no-feedback or endpoint group) were significant, we used a repeated-measurements ANOVA with significance level 0.05. If the null hypothesis was rejected, multiple comparisons using Tukey’s Honest Significant Difference (Tukey’s HSD) method were performed to identify which conditions were significantly different from each other.

Secondly, the performances of groups were compared to each other. The null hypothesis was that there is no difference between performances of participants from different groups under the same condition. To test this, we performed four two-sample t-tests (one for each condition) with Bonferroni corrected alpha to compare performances between the feedback- and no-feedback group. As described above, the endpoint group’s translation condition used the same robot arm position as the BCI condition, but without rotation. When comparing the feedback group with the endpoint group, the endpoint group’s translation condition is therefore compared with the feedback group’s BCI-condition rather than the feedback group’s translation condition.

Lastly, participant’s ratings of the tracking quality (see above) were compared using a Mann–Whitney-U-test with significance level 0.05.

All statistical analyses were performed in Matlab, version 2018a (The MathWorks Inc., Natick, Massachusetts, USA).

## Results

**Figure 4:**
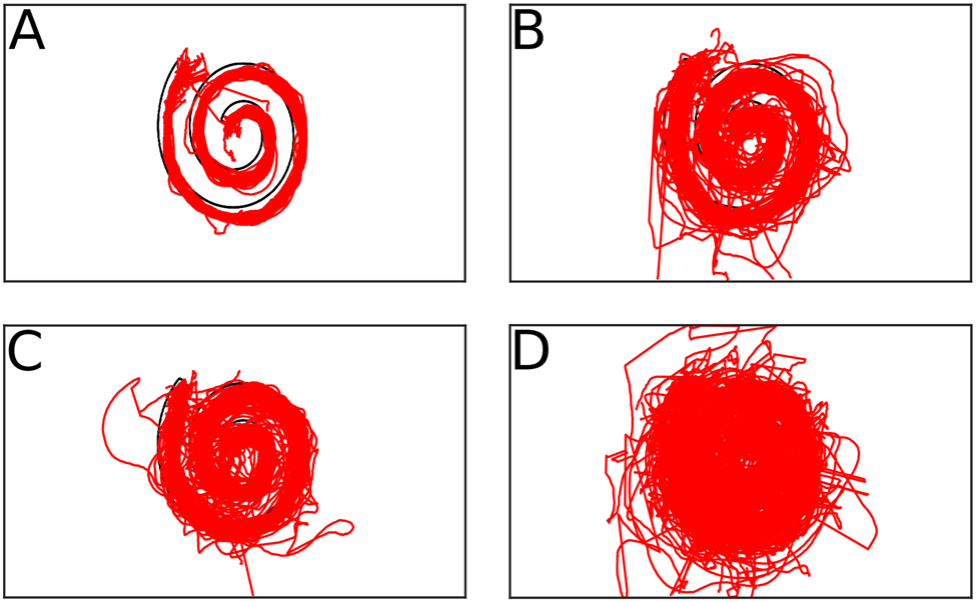
The virtual pens 2D-position on the canvas. The lines participants drew during trials with the spiral shape as a target are shown in red, and the target shape is shown as a black outline for comparison. (**A**)–(**D**) show trials depending on their condition: Ctrl (**A**), translation (**B**), BCI-simulation (**C**) and T + R (**D**).

For a general overview of the data, Fig. 4 shows the 2D tracking data of all participants during ‘spiral shape’ trials as trajectories. Each tracking point represents a two-dimensional position of the pen’s drawing tip on the canvas during the experiment. The data shows that in control condition (Fig. 4a) participants mostly stayed within the outline of the target shape. In the translation and BCI-simulation conditions (Fig. 4b, c) more points are outside of the target shape outline as compared to control. The T + R condition (Fig. 4d) shows the biggest spread of points and many points outside the target shape outline.

**Figure 5:**
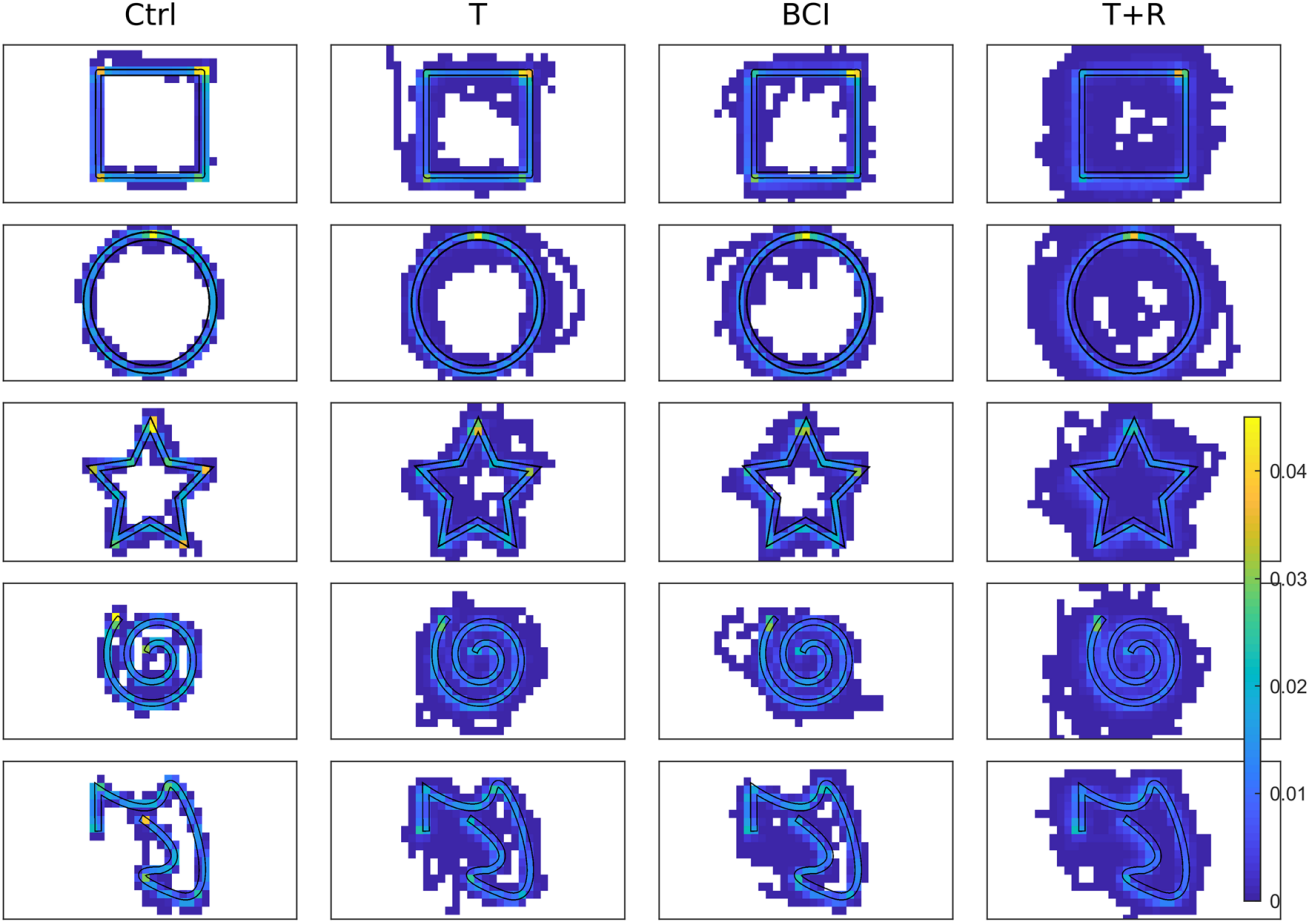
Heatmap-analysis of all shapes and conditions. For each shape (rows) and each condition (columns), all participants’ relative amount of tracking points was averaged and color-coded on a 40 × 20 grid. Cells with a relative amount of exactly 0 tracking points are shown in white, all other values are shown on a scale from blue (low) to yellow (high). Due to a generally higher density of tracking points around the starting area (see text), color values there (and only there) are not to scale.

For a more quantitative analysis of the tracking data, Fig. 0.5 shows the virtual canvas divided into a 40-by-20 grid (grid cells were 2.2 × 2.2 cm in size). For each trial, the number of tracking points within each grid cell relative to the total amount of tracking points in that trial was computed. The mean result of this analysis (over all participants) is shown in Fig. 5. The relative density of tracking points reveals in which segments of the target shape participants spent more time than on others. All heat maps show a high relative amount of tracking points at the shapes starting position (compare Fig. 1), indicating that participants waited at the starting position before beginning their movement. For the control condition, the analysis shows a sharp line along the target shape, with the amount of tracking points outside of the target shape being low. The translation and BCI-sim conditions show a high amount of tracking points along the target shape, but more tracking points outside the boundaries have been recorded as compared to the control condition. Lastly, the T + R condition shows a high amount of data points outside the target shape outline. The relative amount of tracking points appears to be evenly spread across the target outline and the space between the spiral windings. The heat maps for the star and square shapes show a higher relative amount of tracking points in corners than on the linear segments between them, indicating that people spent more time on the corners of these shapes. The heat maps of the asymmetrical shape show a similar result, for example on the high-curvature segment near the end.

**Figure 6:**
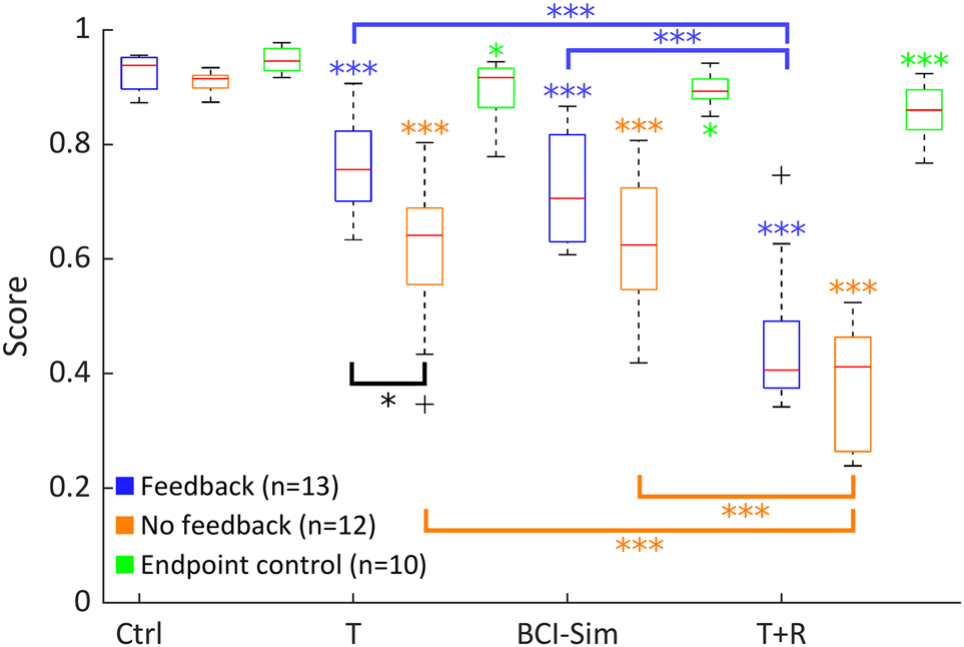
The mean score participants achieved across shapes in the different conditions: Control (Ctrl), Translation (T), BCI-Simulation (BCI-Sim) and Translation plus rotation (T + R). The results are shown for the with-feedback group (blue boxplots), which did the experiment with a physical table matching the virtual tables height, the no-feedback (orange boxplots), which performed the experiment without haptic feedback, and the endpoint group (green boxplots), which did the experiment with a cartesian endpoint control scheme. Stars above boxplots indicate significant differences from the respective group’s control while the blue and purple brackets indicate additional significant differences within their respective group. The black bracket indicates a significant difference between feedback and no-feedback, but within conditions. Note that all conditions (except control) of the endpoint group are significantly different to their counterparts in the feedback-group (not shown in figure).

While the trajectories and heatmaps provide an overview over the raw data, the scoring algorithm (as presented in the methods section) can be used to gain insight into the performance of individual subjects and groups. To compare all participants’ performances in the different conditions in more detail, we computed the mean score of each participant in each condition. The results of participants in the feedback group are shown as blue box plots in Fig. 6. Comparing these results among conditions shows the effects of the different end-effector positions independently of the feedback-error. Using a repeated-measurements ANOVA, we found the difference between conditions to be significant (F(3, 48) = 18.52, *p* = 3.18 × 10^−7^). The data for the control condition shows that people achieve a median score of 93.8% with a maximum score of 95.5% and a minimum of 87.3%. The control condition also shows the smallest spread as compared to the other conditions. Post-hoc comparisons using Tukey’s Honest Significant Difference revealed a significant difference between the control condition and all other conditions (vs. T: *p* = 1.53 × 10^−4^; vs. BCI-Sim: *p* = 3.10 × 10^−6^; vs. T + R: *p* = 3.77 × 10^−9^). In the translation condition, participants reached a median performance of 75.6%, with the variance being higher than in control condition (min: 63.3%, max: 90.6%). In the BCI-sim condition the median score was 70.6% and a similar minimum and maximum as in the translation condition were found (min: 60.8%m max: 86.7%). The post-hoc comparison did not show a significant difference between the BCI-sim condition and the translation condition (*p* = 0.664). The T + R condition had the lowest median score of all conditions (40.7%). The lowest scoring study participant in this condition achieved a score of only 34.2%. However, the highest scoring participant achieved a score of 74.7% in this condition. The post-hoc comparisons revealed a significant difference between the T + R condition and all other conditions (vs. T: *p* = 4.11 × 10^−9^; vs. BCI-Sim: *p* = 2.22 × 10^−8^).

In the following section, we will be comparing the scoring results of the feedback group, which did the experiment with a physical table, to the results of the no-feedback group, which did the experiment without a physical table being present. The data is shown in Fig. 6, where the blue box plots represent the feedback group’s results and the orange box plots show the no feedback group’s results. Pairwise comparisons using Bonferoni-corrected t-tests revealed a significant difference between performance with- or without table only in the translation-condition. (Ctrl: *p* = 0.2272; T: *p* = 0.0031; BCI-sim: *p* = 0.0453; T + R: *p* = 0.0929). However, the no-feedback group’s data shows the same significant differences between conditions that were described for the first group above (rANOVA: F(3,44) = 19.18; *p* = 3.89 × 10^−7^). In the no-feedback group, the difference between control versus translation is significant with *p* = 1.17 × 10^−7^, control vs. BCI-sim with *p* = 2.24 × 10^−7^ and control vs. T + R with *p* = 3.77 × 10^−9^. As for the first group, no significant difference has been found between translation and BCI-sim condition (*p* = 0.9973) while the T + R condition is significantly different to all other condition (vs. T: *p* = 1.17 × 10^−5^; vs. BCI-sim: *p* = 6.06 × 10^−6^).

The results of the endpoint group are shown as green boxplots in Fig. 6. Participants of the endpoint group have generally achieved much higher scores as compared to the other two groups, ranging from 76.8% (T + R Condition) to 97.7% (control). While the results of all conditions in this group are significantly different from control (F(3,36) = 5.5654, *p* = 0.0048, vs. T: *p* = 0.0231 ; vs. BCI: *p* = 0.0415; vs. T + R: *p* = 1.43e−4), none of the conditions are significantly different from each other (T vs. BCI: *p* = 0.9948 ; T vs. T + R: *p* = 0.2840 ; BCI vs. T + R: *p* = 0.1868). When compared to the feedback-group, which did the experiment with rotated end-effector kinematics, we find no significant difference in the control condition using a Bonferoni-corrected t-test (*p* = 0.0588). However, the differences in all other conditions are highly significantly compared their counterparts in the feedback group (BCI: *p* = 1.2151 × 10^−5^; T + R: *p* = 5.0799 × 10^−9^). As mentioned in the methods section, the translation condition of the endpoint group was different from the other groups. If compared to its closest match, the feedback-group’s BCI condition, the difference is highly significant with *p* = 4.0906e−05.

**Figure 7:**
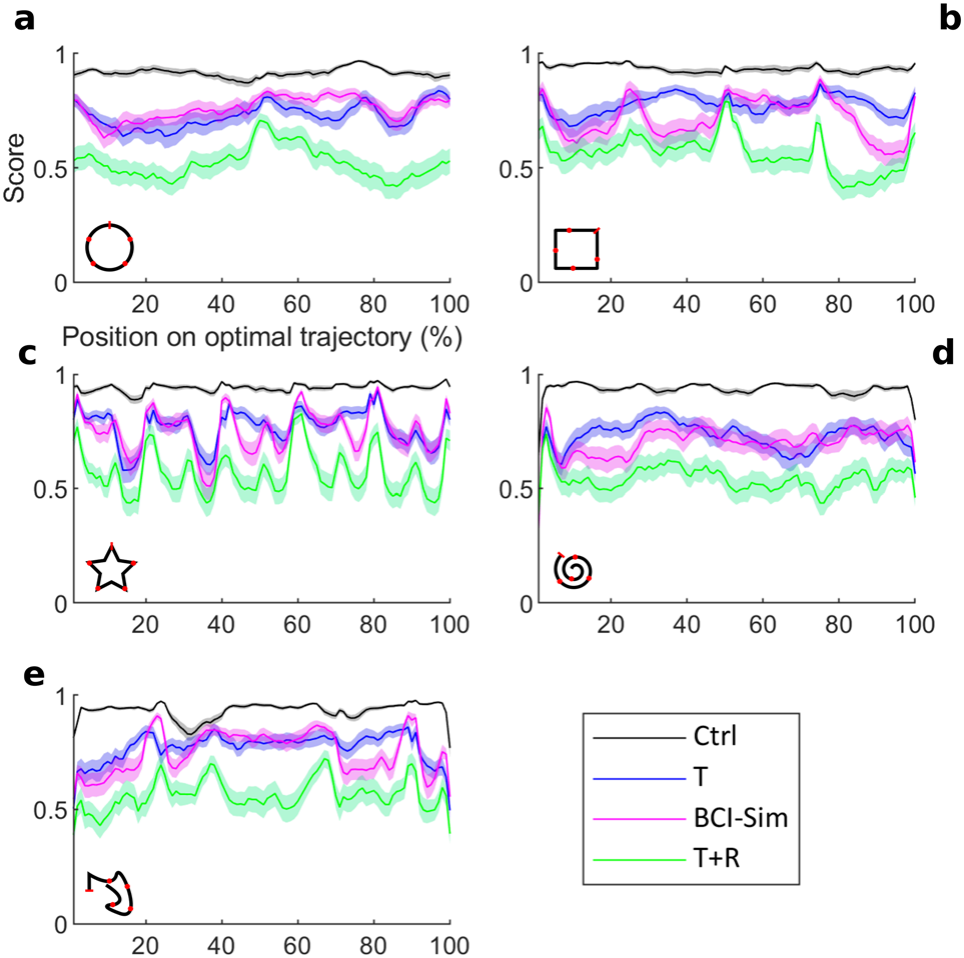
All participants’ mean scores across the target shape. The mean score is shown against the position on the optimal trajectory in percent (see Material and Methods). The data is shown for the Circle (**A**), Square (**B**), Star (**C**), Spiral (**D**) and the custom asymmetrical shape (**E**). For each plot, a symbol indicating the shape is shown in the lower left corner. The respective shapes starting position is marked with a red bar in these symbols. Scores are shown in black for control condition, blue for translation condition, magenta for BCI-Sim and green for T + R condition. The error indicates S.E.M.

The analysis by condition and group does, however, not take into account how participants performed on the different shapes or even particular parts of the shapes. Figure 7 shows the participants’ mean score on segments of the target shape, rather than the mean of a whole trial (see methods section). This analysis shows additional information about how close tracking points within the target outline are to the optimal trajectory. This analysis could reveal whether the performance differences between conditions can be observed everywhere on the target shape or whether they are caused by stark differences in certain features of the target shape (for example, corners). For the circle shape (Fig. 7a), this analysis reveals that participants achieved a higher score in the middle of the shape, particularly in the T + R-condition. The square shape (Fig. 7b) shows a higher score around the corners of the square as compared to the segments between them. This can also be seen in the star shapes analysis in Fig. 7c. Similarly, the asymmetrical shape (Fig. 7e) shows a higher score on corners and segments with high curvature. Lastly, the spiral shape (Fig. 7d) shows a high score at the starting point and a constant performance throughout the rest of the shape. Taken together, participants tend towards a higher score on corner segments as compared to linear segments or segments with a constant curvature.

### Questionnaire results

Table 1 shows how study participants’ rated the difficulty of the shapes in the questionnaires. The response of one subject to this question was invalid and was therefore not considered in the analysis. Questionnaire results across all participants show that people rate the circle shape the easiest with a mean rating of 1.88 (with one being the easiest). Similarly, the square shape was rated 2.03 on average, making it the second easiest shape. The star shape was, on average, rated the highest difficulty (mean 4.5), with 21 participants rating it the hardest and eight participants rating it the second hardest.

**Table 1:** Study participant’s responses in the questionnaire.

Shape	1 (easiest)	2	3	4	5 (hardest)	Mean
Circle	13	16	2	3	0	1.86
Square	13	9	9	3	0	2.03
Star	0	1	3	8	22	4.51
Spiral	2	5	12	9	6	3.34
Asymmetrical	6	3	8	11	6	3.22

Participants were asked to assign a number from one to five to each shape in ascending order of difficulty. The table shows how many participants assigned each number to each shape.

When asked to rate the difficulty of the conditions (except control), participants rated the translation condition the easiest, with a mean of 1.30. Since the translation condition differed in the endpoint group, their results will be discussed separately below. In total, 18 out of the 25 participants in the feedback- and no-feedback group rated the translation condition the easiest and no one rated it the hardest. The CI-sim condition had a mean rating of 1.78 with seven participants rating it the easiest and two participants rating it the hardest. Apart from these two participants, the other 21 participants rated the T + R condition the hardest, with no one rating it the easiest, resulting in a mean rating of 2.91 for this condition. In the endpoint group rated the BCI condition and their version of the translation condition (which differed only by the missing rotation) equally difficult, with a mean of 1.78. However, six out the ten participants of this group rated the T + R condition the hardest, resulting in a mean score of 2.44.

The feedback group, which did the experiment with a physical table being present, rated the tracking quality with a mean of 4.08 on a five-point Likert scale. Only two participants in this group rated the tracking quality to be medium (3) and no one rated it lower than that. The no-feedback group, performing the experiment without a table, rated the tracking quality with a mean of 3.67. Six participants in the no-feedback group rated the tracking quality to be medium and no participant rated it lower. However, only two participants from the no-feedback group were rating tracking quality with the highest possible rating. The differences in tracking quality rating between the groups is not significant (Mann–Whitney U-test; *p* = 0.085). The endpoint group rated the tracking quality with a mean of exactly four, with one rating each for three and five. Compared to the feedback group, the ratings of the endpoint group are not significant (*p* = 0.7596).

## Discussion

The main goal of this study was to investigate the importance of end effector positioning and indirect haptic feedback on participants’ performances in a virtual reality model of a BCI-scenario. Most invasive BCI studies are single-case studies and their results are usually not well comparable due to differences in the hardware, the decoding and the tasks used. The performance of BCI-systems has, for example, been measured using the Action Research Arm Test (ARAT;^2,21^), a clinical test often used to assess extend of upper limb motor impairments in patients with stroke, brain injury or similar conditions. While these tests could provide a comparison of patients using a BCI with healthy subjects, they are still limited by the number of available patients enrolled in BCI studies and by the fact that typically not all ARAT subtests are suitable for BCI use. In our VR-based model, healthy participants experienced a situation that is similar to what a tetraplegic patient would experience using an invasive BCI system. This allowed us to record data from a bigger group of participants in a controlled environment. Investigating the feedback error, we found a significant effect of lacking indirect haptic feedback only in the translation condition. Regarding the alignment error, our data suggests that every misalignment of the end-effector leads to a significant loss of performance regardless of the control scheme used. However, the global cartesian endpoint control scheme has a much lower (~ 3%) impact on motor performance compared to a situation where the end effector kinematics are rotated. In the feedback group, even in the translation condition (where participants scored highest) we found a 15% loss of performance compared to control. Under both control schemes, the highest loss of performance was found when the end-effector was rotated by 90 degrees relative to the participants (T + R-condition).

### Method discussion

The shape drawing task we used in this experiment is similar to the mirror drawing task, a widely used neuroscientific method to assess motor performance and -learning. It has, for example, been used to evaluate the motor learning capabilities of patient H.M. in the early 1960s^22^ and is used to this day in both science^23^ and clinical diagnostics^24^. However, we decided to lower the difficulty by using bigger shapes and not strictly enforcing the time limit. This was done to ensure that the task remained solvable under difficult conditions and remained rewarding for the participants.

Previous research has shown that participants perform worse in fine motor tasks in VR as compared to a real-world version of the same task^25^. In contrast, participants in our task achieved near-optimal results in the control condition (compare Figs. 6, 7). It could be argued, however, that the difference in motor performance caused by VR was too small to be captured in our results since we used bigger shapes. However, any performance loss due to the usage of VR would affect all groups and conditions equally, since the hardware used and the VR environment remained unchanged among conditions. If a similar experiment would be performed without VR, the size of the target shapes and the time limits would probably still have to be adjusted. Additionally, a non-VR version of this task would pose the additional problem of holding the pen firmly over the course of the experiment, which can be problematic in BCI scenarios. A test with a clinical population using a BCI-controlled robot arm would, however, provide the most reliable data about the optimal end-effector positioning and the performance loss that can be attributed to it.

Participants’ near-optimal results under control condition provide evidence that there was no error in translating participants’ movement intentions into our simulated BCI-system. In a real-world BCI experiment, this would relate to an ideal decoder. This allowed us to study the alignment error and the feedback error, as defined in the introduction, independent from any decoding error. Additionally, we can compare the performance of the feedback group under control condition to the performance of the no-feedback group under control condition, allowing us to investigate the feedback error independent from both the decoding- and the alignment error. It could be argued, however, that the VR setup and the virtual avatar represent a source of alignment error even under control condition. If that is the case, the influence of the alignment error would still be equal for all groups, leaving the missing somatosensory feedback the only difference between them.

### Analysis of feedback error

Recent BCI experiments investigated the introduction of artificial somatosensory feedback by providing touch information gathered by sensors built into the end-effector back to the brain using functional stimulation^9,10^. Similarly, it has been shown that the use of a multisensory prosthesis, which provides somatosensory feedback via peripheral nerve stimulation, reduces distorted phantom limb perceptions in amputees^26^. With the no-feedback group in our experiment, we aimed to investigate the effects of missing or incomplete somatosensory feedback on participants’ performances. During our experiment, participant’s proprioceptive feedback remained unaltered. However, we were able to manipulate the indirect haptic feedback that participants received when drawing on the virtual table by either using a real table or not. We argue that the embodiment of the virtual robotic arm was more difficult for no-feedback group than for the feedback group. However, removing the indirect haptic feedback when the virtual pen touched the virtual table did not lead to a significant effect on participants’ performances (Fig. 6) under control conditions. A study investigating a patient with a deafferented arm found that the patient had a degraded handwriting and severe problems when drawing an ellipsis, particularly with eyes closed^27^. Such a combined effect of impaired visual and tactile feedback might explain the significant difference of lacking indirect haptic feedback in the translation condition, since the translation condition is the condition where we expected the visual observation of the robot arm’s movement to be the worst. In contrast to the study by Teasdale et al. (mentioned above), the somatosensory feedback of the participants’ hands touching the controller was unaffected in our experiment. However, since this feedback does not change during the task and the feedback about merely holding the controller does not provide information that is relevant to the task, we argue that this does not considerably affect participants’ performances. The indirect feedback inferred from the controller touching the table is the significant source of feedback in this experiment. This means participants in our study were not completely deprived of somatosensory feedback but were only missing a significant part of it. On the other hand, it should be noted that the patient investigated by Teasdale and colleagues also had impaired proprioception, which was not the case for participants in our experiment. This could explain the comparably small effect of incomplete somatosensory feedback on participants’ performances in the data presented here. With the intact proprioceptive feedback of our participants and the unaffected sense of holding the controller in hand, our experiments show that even small inconsistencies between modalities can negatively affect motor performance.

In part, the performance difference between the feedback and the no-feedback group might be attributed to the mismatch of proprioceptive information and the visually observed position of the end-effector. While this seems to have a negligible effect on performance under control condition, it could be argued that this effect becomes increasingly important for conditions with misaligned end-effectors. Since visual, haptic and proprioceptive feedback has been shown to be integrated and plays an important role in motor performance^28^, It could therefore be argued that controlling a robot arm with unaffected proprioception like in our experiment might be more difficult than a BCI setup without proprioceptive feedback. On the other hand, a complete lack of proprioception can significantly affect motor performance (as discussed above).

Combined, these findings could hint towards a particularly high importance of haptic feedback in grasping and object interaction tasks, where other experiments have shown that the availability of somatosensory feedback has a significant impact in invasive BCI scenarios^29^. Our data shows smaller effect of incomplete somatosensory feedback on motor performance, which could indicate that following trajectories might depend less on indirect haptic feedback. However, since the performance loss that can be attributed to the feedback error varies among conditions, the data presented here provides evidence that a combination of suboptimal vision and impaired feedback maximizes the impact on motor performance, and that the effects of alignment- and feedback error are therefore intertwined. These results also highlight the important role of the combined feedback strategy and the sensory encoding of artificial feedback, as even slight impairments of somatosensory feedback can cause significant differences in motor performance.

### Analysis of alignment error

With the different conditions we tested in the experiment, we were able to induce different strengths of alignment-error and study their effects independent from the other error sources. As described in previous research, higher rotation angles have a bigger impact on motor performance in visuomotor adaptation experiments^6^. By testing several rotation angles (no rotation, 45 degrees and 90 degrees), we tried to provide data that can inform future decisions about optimal robot arm positioning in BCI scenarios. Additionally, the inclusion of the endpoint group allowed us to compare motor performances of different control schemes in our study.

Our data suggests that with the rotated end-effector kinematics of the feedback and no-feedback groups, the T + R condition has a significantly larger impact on participants’ performances than the other two conditions. This is in line with previous research, where Cunningham and colleagues found the lowest performance in 2D visuomotor rotation tasks for rotation angles between 90° and 135°^6^. Additionally, participants in our study rated the T + R-condition with its 90-degree rotation the hardest. In the endpoint group, participants also had the lowest mean performance under the T + R condition, even though no significant difference was found compared to the other two. In the translation and the BCI-simulation condition we see a smaller effect on participants’ performances, with no significant difference between them in any group. However, participants’ responses in the questionnaires raise the question whether there are individual differences as to which condition is considered easier.

We initially expected a trade-off between how strong the robot arm is rotated and how well the participant was able to visually observe the robot arm’s movements. In the T + R-condition, the robot arm can be easily observed and operates within the participant’s peripersonal space, but the strong rotation requires intense adaptation. The translation condition marks the opposite extreme in this trade-off, with no rotation to adapt to, but suboptimal visual observation due to the sideward translation. Interestingly, the endpoint group still had the worst mean performance under T + R condition, even though the end effector kinematics are not rotated and vision is very similar to control condition. This result indicates that the visuomotor rotation and visual feedback are not the only factors affecting the performance in these scenarios. We can infer from our data that the optimal positioning of the end effector probably lies in the range between the translation and the BCI-simulation condition, while some individual variance cannot be excluded. This is supported by research performed by Risso and colleagues, who have shown in a single-case study that integration of both visual and somatosensory information significantly shapes perception^30^. Future research should therefore cover more combinations of rotation angle and displacement to further advise the robot arm placement in BCI-scenarios. Additionally, different robot arm placements and rotation angles might differ in the time participants need to adapt to, which could be another factor in deciding for an optimal robot arm placement in BCI setups.

Additionally, we compared motor performances under two different control schemes: In the relative coordinate frame control scheme that was used with the feedback and no-feedback group, the end-effector kinematics were rotated in the same way as the robot arm. In contrast, the endpoint-group used a global cartesian coordinate frame control scheme, where the movement endpoint position was kept relative to the participant, independent of the robot arm rotation. A forward movement using the relative coordinate frame was therefore translated into a movement in the end-effector’s forward direction (including its rotation), while under the global cartesian coordinate frame the direction would always stay relative to the participant. The former control scheme resembles a BCI setup where no additional transformation of the endpoint is performed^3^ or the control signal is decoded in joint-space rather than a three-dimensional endpoint^31,32^. Both systems have been recently compared in a BCI study^33^ that found endpoint control to be generally preferable, even though a combination of both control schemes has been shown to be effective in the same study. Our data shows a similar result, with the cartesian endpojnt control scheme having a much smaller impact on motor performances. However, motor performances remain significantly different from control condition, indicating that this control scheme is not entirely free of alignment error either. However, the global cartesian endpoint control scheme in our experiment reduced the effects of different robot arm rotations on motor performances so that no significant differences between the conditions could be found anymore, further supporting the conclusion that this control scheme should be preferred for BCI control.

However, our results show that participants’ performances significantly decrease as compared to control condition when controlling the robot arm. Even the condition participants performed best in (translation) had a median score ~ 15% lower than control when the end effector kinematics were rotated and ~ 3% lower when a cartesian endpoint control scheme was used. Given that this loss of motor performance occurred within our model of an ideal decoder, we predict that completely eliminating the alignment error (for example by using an exoskeleton or functional electrical stimulation) would significantly increase the performance of patients using an invasive BCI.

Other studies found results similar to ours in situations where the participants’ viewing angle was rotated relative to a tool they use, for example in surgical environments. A study by Wentink and colleagues^7^ investigated physicians’ performances in simulated endoscopic surgeries and found a significant effect of similar size when the physicians observed their instruments movement from a camera angle rotated by 90 degrees. Similarly, Klein and colleagues studied novice physicians’ performances in a laparoscopic surgical environment^8^. They also found the worst performance when the instrument was rotated by 90- or 135 degrees relative to the surgeon, a result in line with the basic research by Cunnigham^6^. While these studies focus on the effects of rotated visual feedback on tool use, we argue that suboptimal embodiment of the robot arm contributes to the performance loss found in our experiment as an additional component. This is supported by another line of evidence surrounding the rubber hand illusion experiment^34^. A variant of this experiment has been expanded to include a motor component by having a finger of the rubber hand move along with the same finger of the participant’s hand^35^. the added sense of agency was found to strengthen the illusion, indicating that the sense of ownership over an object can be enhanced by motor control. As mentioned above, a patient using a BCI would ideally include the robot arm in their body scheme and develop a sense of ownership and agency towards it. The rubber hand illusion shows that this could be possible. However, this kind of embodiment has been shown to depend on the position of the rubber hand relative to the real hand. Previous work done by Perez-Marcos and colleagues^36^ showed that embodiment of the rubber-hand^34^ was significantly impaired when the rubber hand was placed in a physiologically implausible angle relative to the participant. While it could be argued that all three conditions tested in our study are physiologically implausible due to the distance from the participants’ bodies, this indicates that in the T + R-condition, apart from the visuomotor misalignment caused by the rotation, a missing embodiment and impaired sense of ownership of the robot arm might contribute to the performance loss we see in the data. The results of the endpoint group support this view. The high performance loss in the T + R condition compared to the other conditions could be attributed to an impaired embodiment, offering an explanation for the strong performance loss despite better a better view of the working space and no visuomotor rotation. However, the high performance loss seen in the feedback group’s translation condition (which resembles the endpoint control scheme) shows that visual observation of the working space still can have a very large impact on motor performance. Combined, our results confirm that robot arm embodiment is significantly contributing to motor performance in BCI scenarios and that the end-effector positioning plays an important role in the control of the end effector. Future research about end effector embodiment in BCIs might also focus on the robot arms visual appearance, with more anthropomorphic arms allowing for a better embodiment, as research by Maimon-Mor and Makin have shown^37^.

In summary, the data presented in this work gives an insight into the effects of deprived somatosensory feedback on fine motor performance in BCI scenarios. Our analysis shows that the lack of indirect haptic feedback alone had a minor impact, but that the effect was amplified with alignment error as an additional error source in place.

As for the alignment error, our data shows the impact of end-effector positioning on motor performance in BCI-scenarios and provides evidence that the optimal position of the end-effector might vary individually. Our results also indicate that a natural positioned effector device could significantly benefit how patients using an invasive BCI perform when controlling a robotic arm. Our study therefore highlights the importance of optimal positioning and embodiment of end-effectors and developing end-effectors that allow for a better embodiment and a more natural positioning, i.e. exoskeletons or functional muscle stimulation^18,19^.

## Supplementary Information

Supplementary Figure S1.

## Data Availability

The datasets generated and analysed during the study reported here are available from the corresponding author on reasonable request.
