# Supplementary material for: Quantifying the alignment error and the effect of incomplete somatosensory feedback on motor performance in a virtual brain–computer-interface setup
Source: Sci Rep. 2021 Feb 25;11:4614. doi: 10.1038/s41598-021-84288-5 (PMC7907076; doi:10.1038/s41598-021-84288-5)
Supplement: Supplementary file 1 — Supplementary Figure S1. [file 41598_2021_84288_MOESM1_ESM.pdf]

Quantifying the alignment error and the effect of incomplete somatosensory feedback on motor performance in a virtual brain-computer-interface setup

Robin Lienkämper[1*], Susanne Dyck[1], Muhammad Saif-ur-Rehman[1], Marita Metzler[1], Omair Ali[1], Christian Klaes[1]

Supplemental Material

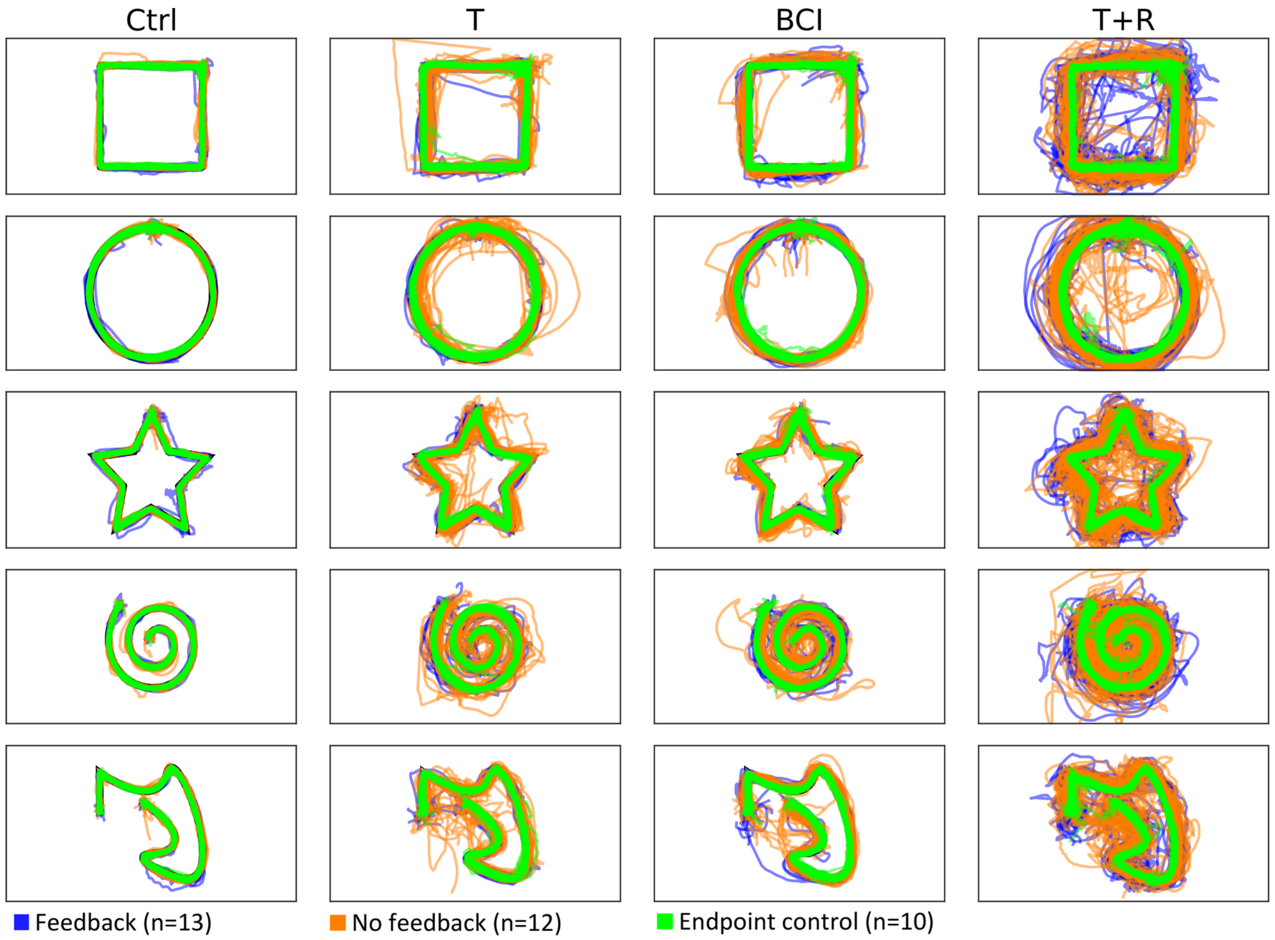

Supplemental Figure 1: The virtual pens 2D-position on the canvas, shown for of all participants and all target shapes. Trajectories of the feedback group are shown in blue, those of the no-feedback group in orange and the endpoint group in green.
